# Dental Casts for Fixed Dental Prostheses Printed with SLA Technology: Influence of External Shell Thickness and Printing Orientation

**DOI:** 10.3390/ma18102246

**Published:** 2025-05-12

**Authors:** Ignacio García-Gil, Verónica Rodríguez Alonso, Celia Tobar Arribas, Seyed Ali Mosaddad, Jesús Peláez, María J. Suárez

**Affiliations:** 1 Department of Conservative Dentistry and Bucofacial Prosthesis, Faculty of Odontology, Complutense University of Madrid, 28040 Madrid, Spain; garciagil.ignacio@gmail.com (I.G.-G.); veranicr@ucm.es (V.R.A.); cetobar@ucm.es (C.T.A.); semosadd@ucm.es (S.A.M.); mjsuarez@ucm.es (M.J.S.); 2 Department of Research Analytics, Saveetha Dental College and Hospitals, Saveetha Institute of Medical and Technical Sciences, Saveetha University, Chennai 600077, India

**Keywords:** 3D printing, accuracy, dental casts, external shell thickness, print orientation, stereolithography

## Abstract

Printed cast models are becoming increasingly important in prosthodontics. The aim of this in vitro study was to evaluate the influence of print orientation and external shell thickness on the accuracy of stereolithography (SLA) master casts for fixed dental prostheses. Seventy-two maxillary hollow master casts were fabricated from a standard tessellation language (STL 0) reference file containing dental preparations. The casts were divided into six groups (n = 12 per group) according to internal shell thickness (2 mm and 4 mm) and print orientation (0°, 10°, and 20°). Discrepancies between STL 0 and the STL files of the printed casts were measured using the root mean square (RMS) error. Data were statistically analyzed using a one-way Kruskal–Wallis test to assess trueness, and precision was evaluated with the Levene test (α = 0.05). No statistically significant differences were found in any of the tested conditions. Print orientation and cast thickness did not influence the overall accuracy of SLA master casts for fixed dental prostheses.

## 1. Introduction

Computer-aided design and computer-aided manufacturing (CAD-CAM) technologies, together with advances in digital materials and workflows, have initiated a digital revolution in dentistry [1,2,3]. With modern CAD softwares, dental technicians and restorative dentists can analyze standard tessellation language (STL) files generated from intraoral scans, enabling the virtual design of various dental devices such as diagnostic casts, treatment casts, crowns, bridges, and surgical guides [4]. Following the digital design process, the manufacturing (CAM) stage can be performed using either subtractive or additive techniques, depending on the clinical indication and material requirements.

Subtractive manufacturing involves the mechanical removal of material from solid blocks using various tools in either dry or wet conditions. While this approach enables the fabrication of complex geometries in ceramics, metals, and polymers, it is associated with high costs and substantial material waste [5,6,7]. As an alternative, additive manufacturing (AM), also known as 3D printing or rapid prototyping, has gained significant attention in recent years. AM technologies construct complex geometries by sequentially adding material, thereby minimizing waste and improving production efficiency [8,9,10].

In contemporary dental practice, a broad spectrum of materials—including ceramics (such as zirconia and lithium disilicate), metals (like titanium and cobalt–chromium alloys), and various polymers—are selected based on the specific requirements of prosthetic and restorative treatments. Of particular relevance to digital workflows are photopolymerizable resins, which have become the material of choice for AM technologies. These resins allow for the efficient and precise fabrication of diagnostic casts, master casts, surgical guides, temporary crowns, fixed dental prostheses (FDPs), splints, try-in, or individualized trays [1,4,11,12]. Of the seven categories of 3D printing defined by the American Society for Testing and Materials (ASTM), stereolithography (SLA), material jetting (Multijet), and material extrusion, also known as fused deposition modeling (FDM), are the most commonly employed technologies for the fabrication of dental casts [13].

Objects printed using SLA technology are produced layer by layer from a vat of photopolymerizable liquid resin, into which a build platform is immersed [14]. This category encompasses both SLA, which utilizes ultraviolet (UV) laser light to cure the resin, and digital light processing (DLP), which employs a digital light projector as the curing source. The primary distinction between these techniques is that SLA traces and cures each cross-sectional area individually, whereas DLP simultaneously projects and cures the entire cross-section of the object [7]. In contrast, the technologies employed by Multijet and FDM differ substantially. Multijet also utilizes UV light to polymerize droplets of material dispensed from multiple nozzles, while FDM creates objects by extruding a thermoplastic filament through a heated nozzle, building the object layer by layer from the bottom up [14,15,16,17].

The different 3D printing technologies, as well as the type of resin, the geometry of the design, the printing orientation, the thickness of the layer, the number and position of support structures of the printed cast, and, where applicable, post-processing after printing are factors that seem to influence the accuracy of dental physical casts [18,19,20]. Accuracy of a 3D printer is defined as the combination of two parameters: trueness and precision, according to the International Organization for Standardization (ISO) [21]. Trueness refers to the degree of deviation between the printed object and the original reference model, while precision indicates the degree of variation among multiple printed objects produced under identical conditions [22,23]. In dental applications, achieving both high trueness and high precision is essential to ensure proper fit, functionality, and long-term success of prosthetic restorations. Print orientation is a key factor affecting both the dimensional accuracy and surface characteristics of 3D-printed dental casts; recent studies [20,24,25,26,27,28] show it can significantly influence properties like surface wettability and mechanical performance. Shell thickness is equally important, as thicker shells generally improve structural rigidity and resistance to deformation but may increase material usage without always enhancing accuracy. In contrast, thinner shells are more cost-effective but can be prone to distortion or warping during post-processing. Therefore, optimal shell thickness should achieve a balance between mechanical stability and dimensional accuracy [25,29,30,31,32].

Despite the recognized importance of both print orientation and shell thickness, there is a scarcity of studies specifically analyzing their combined effects on the accuracy of master casts intended for FDP applications [32,33,34,35,36]. Therefore, the aim of this in vitro study was to evaluate how print orientation (0°, 10°, and 20°) and outer wall thickness (2 mm and 4 mm) influence the accuracy of SLA-printed master casts fabricated with a dental desktop printer. The null hypothesis was that no differences would be found in the accuracy (trueness and precision) values of the SLA master casts manufactured for each of the different situations of print orientations and external shell thickness.

## 2. Materials and Methods

**Figure 1:**
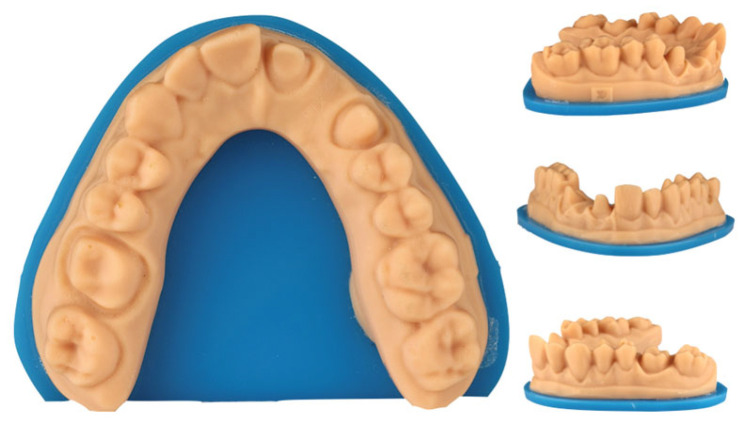
Master cast with dental preparations designed for cut-back zirconia restorations.

Seventy-two printed casts were fabricated from a standard tessellation language (STL 0) reference file representing a maxillary arch prepared for cut-back zirconia restorations. The reference model included a three-unit fixed partial denture (FDP) extending from the left central incisor to the left canine, as well as a single crown preparation on the right first molar (Figure 1). The abutment teeth were prepared with 1 mm of axial reduction, a 1 mm-wide chamfer finish line, a 6° convergence angle, and rounded internal angles. The convergence angle, also referred to as the taper, describes the angle formed between the opposing axial walls of a prepared tooth or abutment. This angle facilitates the proper placement and retention of prosthetic restorations. In this study, a 6° convergence angle indicates that the opposing walls of each abutment are inclined at a total of 6 degrees relative to each other, which is a commonly recommended value to ensure adequate retention and an appropriate path of insertion for FDP. The STL 0 reference file was generated using a dental laboratory scanner (T710; Medit, Seoul, Republic of Korea) with a reported accuracy of 4 µm.

The casts were fabricated using an SLA dental printer (Form 2, Formlabs, Somerville, MA, USA). The printer features a construction volume of 145 × 145 × 175 mm, an XY resolution of 140 μm, and a laser point size of 140 μm, and operates with a laser power of 96 mW at a wavelength of 405 nm. Printing parameters were set to a layer thickness of 50 μm (Z-axis resolution), and print preparation was performed using the PreForm 3.47.0 software (Formlabs, Somerville, MA, USA). A methacrylate-based resin (FotoDent model2 385 nm beige, Dreve, Unna, Germany) was used for all prints. Prior to fabrication, the printer was calibrated according to the manufacturer’s instructions.

**Figure 2:**
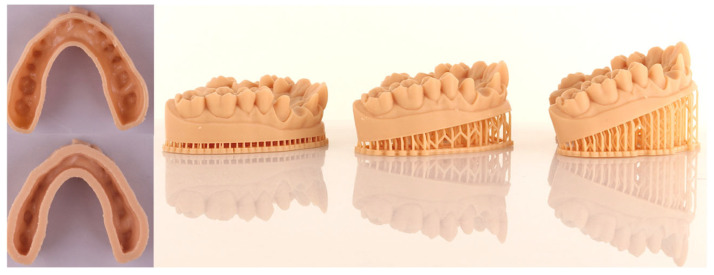
External shell thicknesses (2 mm and 4 mm) and print orientations (0°, 10°, and 20°) of the master cast.

Six groups were established based on the external shell thickness and print orientation of each cast: Group 1 (G1): 2 mm and 0°, Group 2 (G2): 2 mm and 10°, Group 3 (G3): 2 mm and 20°, Group 4 (G4): 4 mm and 0°, Group 5 (G5): 4 mm and 10°, and Group 6 (G6): 4 mm and 20° (Figure 2). Twelve casts were fabricated for each group, as determined by power analysis (effect size = 1.2, power = 80%) performed using statistical software (G*Power 3.1.9.4).

Once all specimens were printed, a standardized post-processing protocol was performed for all casts in accordance with the manufacturer’s instructions. First, each specimen was rinsed with 96% isopropyl alcohol at 20 °C and 50% humidity for 10 min. After drying, the casts were polymerized in a curing unit (Otoflash G171, NK-Optik, Baierbrunn, Germany), which emits light in the range of 280 to 700 nm (10 flashes per second) using two lamps. In this study, each cast was subjected to 2000 flashes, resulting in a post-crosslinking time of 3 min. Finally, all specimens were stored in a darkened room at 23 °C.

**Figure 3:**
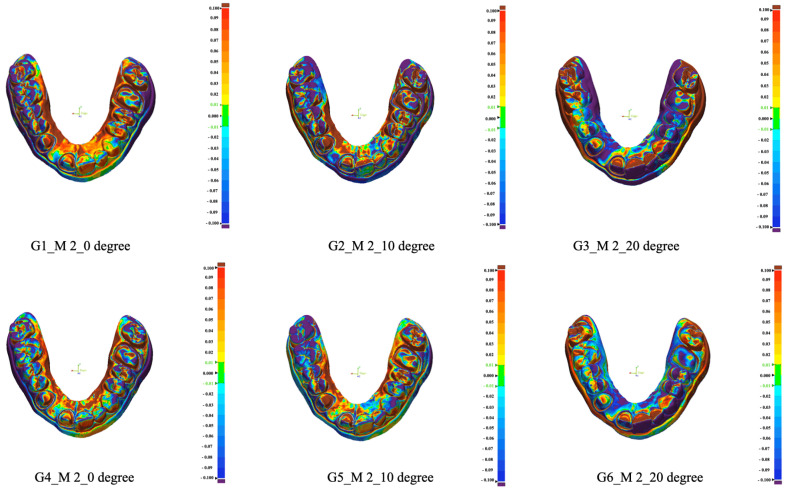
Superimposition of the STL file of each model with the STL 0 reference file for all evaluated groups.

Each specimen was digitized within 48 h of printing using the same dental laboratory scanner (T710) that was used to generate the initial STL file. The scanner was calibrated according to the manufacturer’s instructions prior to data acquisition for each group. Discrepancies between the STL 0 reference file and the STL file of each specimen were determined by calculating Euclidean measurements and root mean square (RMS) error values using reverse engineering software (Geomagic Control X version 2020.0.1; 3D Systems, Rock Hill, SC, USA) (Figure 3), following superimposition of the files.

**Figure 4:**
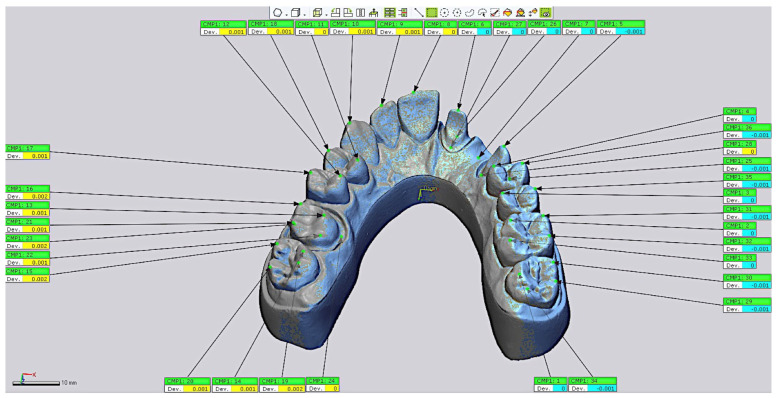
The 36 points marked at critical locations on each cast.

Furthermore, additional measurements were performed to assess discrepancies based on specific locations within each model. For this purpose, 36 points were marked on each cast, corresponding to the cusps of all teeth, the finish lines of the abutment teeth, and the center of the pontic area (Table 1) (Figure 4).

Finally, deviations at the specific points corresponding to the abutment teeth and pontic area (5, 6, 7, 13, 16, 21, 22, 23, 24, 25, 26, 27, and 28) were evaluated to determine whether significant differences existed according to the analyzed axis (x, y, and z) (Table 1). Such deviations could potentially lead to a lack of passive fit in the restoration or issues with occlusal adjustment. The x-axis represents the buccolingual direction, the y-axis corresponds to the mesiodistal direction, and the z-axis denotes the occlusogingival direction of the printed casts.

The following group comparisons were performed to evaluate the effect of external shell thickness on the trueness of the printed casts: G1 vs. G4, G2 vs. G5, and G3 vs. G6. Additional comparisons (G1 vs. G2, G2 vs. G3, G1 vs. G3, G4 vs. G5, G5 vs. G6, and G4 vs. G6) were conducted to assess the influence of print orientation as the second variable. These analyses were carried out both globally for the entire model and at each of the 36 marked points. Trueness was defined as the closeness of the STL file of each scanned specimen to the STL reference file, whereas precision was defined as the standard deviation (SD) within each group [22,23].

The Shapiro–Wilk test indicated that the RMS data for both the total cast and the selected points were not normally distributed (*p* < 0.05). Consequently, the Kruskal–Wallis test was used to analyze the trueness of the total cast and the selected points. Precision was assessed using the Levene test based on the median. All statistical analyses were performed using statistical software (IBM SPSS Statistics, v.26; IBM Corp, Armonk, NY, USA), with a significance level set at α = 0.05.

## 3. Results

Table 2 shows the overall minimum, maximum, and the mean ± SD RMS error discrepancies (trueness ± precision) for each group. Each group consisted of 12 measured samples (n = 12), for a total of 72 specimens evaluated. When minDV and RMS values were analyzed, the Kruskal–Wallis test revealed no significant differences among the groups (*p* < 0.05). However, when G3 was compared with G6 in maxDV value, statistically significant differences (*p* = 0.019) were observed.

When evaluating the effect of external shell thickness on the overall RMS value of the casts, no significant differences were found between the following group comparisons: G1 vs. G4 (*p* = 1), G2 vs. G5 (*p* = 0.1938), and G3 vs. G6 (*p* = 0.1570). Similarly, when assessing the impact of print orientation on the overall cast, group comparisons revealed no statistically significant differences [G1, G2, G3 (*p* = 0.4431); G4, G5, G6 (*p* = 0.2432)].

**Figure 5:**
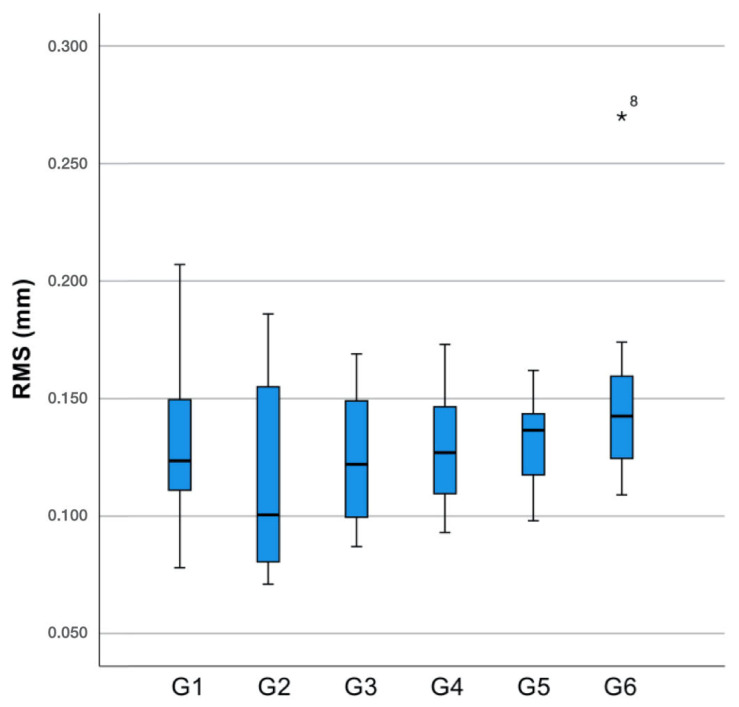
Box plot of overall root mean square (RMS) error discrepancies (mm) for the tested groups. The asterisk (*) denotes an outlier in Group 6 (G6).

The highest trueness value among the analyzed groups was observed in G2 (0.116 ± 0.0421 mm), while the lowest was found in G6 (0.151 ± 0.0424 mm) (Figure 5). The Levene test based on the median was used to evaluate the overall precision of the casts and showed no significant differences among the groups (*p* = 0.432). Group 5 demonstrated the highest precision, whereas Group 6 exhibited the lowest.

For x-axis, y-axis, and z-axis discrepancies, the Kruskal–Wallis test revealed significant differences in trueness when evaluating external shell thickness. Groups with a 2 mm shell thickness demonstrated better trueness compared with those with a 4 mm thickness. In contrast, print orientation did not significantly affect trueness (*p* > 0.05), except at point 2 (*p* = 0.036) (Table 3). Additionally, no significant differences were observed between the points marked in the anterior region and those in the posterior region. Thus, trueness was not influenced by the anterior or posterior location, regardless of the group evaluated.

Finally, discrepancies along the x-axis, y-axis, and z-axis at the selected points corresponding to areas intended for fixed prostheses were evaluated. The Kruskal–Wallis test revealed no significant differences in trueness when assessing the effect of print orientation. However, significant differences were observed when considering the external shell thickness variable (Table 4). Specifically, at the incisal edge and canine cusp points, groups with a 2 mm shell thickness demonstrated superior trueness compared with those with a 4 mm thickness across all axes. Although trueness was generally better in the 2 mm groups at most evaluated points, this finding was not consistent across all axes for every location.

## 4. Discussion

Based on the results of the present study, no significant differences in trueness or precision were observed between treatment casts printed with an SLA 3D printer using different print orientations and outer wall thicknesses. Therefore, the null hypothesis was partially accepted: while statistically significant differences were identified between certain groups at specific measured points, no differences were found at the overall cast level. The trueness of the printed casts ranged from 0.0739 mm to 0.1934 mm, which falls within the clinically acceptable manufacturing range of 0.1–0.3 mm [37,38,39]. Group 2 (SLA, 2 mm thickness, 10° orientation) exhibited the highest mean trueness (0.116 ± 0.0421 mm), whereas Group 6 (SLA, 4 mm thickness, 20° orientation) demonstrated the lowest mean trueness (0.151 ± 0.0424 mm). Although no statistically significant differences were observed between groups at the overall cast level, significant differences were detected at the tooth and point level, particularly between casts printed with 2 mm and 4 mm shell thicknesses.

The accuracy of AM, encompassing both trueness and precision, is determined by multiple variables that can affect the final outcome. In this study, only print orientation and internal wall thickness were evaluated as variables using SLA technology. However, other factors are known to influence the accuracy of printed casts. For example, the duration of post-printing polymerization can impact dimensional stability, as polymerization shrinkage may continue for 3–4 weeks after printing [40,41]. Additional factors such as the amount of resin in different regions of the cast, variations in cast geometry, the speed and intensity of the polymerization laser, support structure design, post-processing procedures, and layer thickness also play significant roles in determining the overall accuracy of 3D-printed dental models [42,43,44,45,46].

The current scientific literature contains relatively few studies addressing the influence of print orientation on the accuracy of dental casts. Short et al. demonstrated in their in vitro study that casts printed with an SLA printer (Form 2; Formlabs) at orientations of 0° and 20° exhibited lower volumetric discrepancies than those printed at 90° [36]. In the present study, print orientations of 0°, 10°, and 20° were selected based on both manufacturer recommendations and previous research, which indicate that these angles optimize trueness and reduce distortion in full-arch SLA-printed dental models [47,48]. Additionally, since our study focused on casts intended for fixed prostheses, where accuracy is critical to achieving passive fit of restorations, these orientations are especially relevant. This observation is corroborated by Maneiro et al., who found that a 22.5° print orientation resulted in the lowest manufacturing discrepancies relative to the original STL, although a vat-polymerization daylight polymer printer (Photon Mono SE, LCD 2K) was used in their work [48]. Shin et al. also employed an SLA 3D printer (Form 3, Formlabs) to produce both full-arch casts at 0° and partial-arch casts at 0° and 60°, using cast thicknesses of 1, 2, 3, and 4 mm. Their results showed a trueness error of 73.60 ± 2.61 μm for full-arch casts at 0°, 49.54 ± 8.16 μm for partial casts at 0°, and 40.66 ± 6.80 μm for partial casts at 60°. Notably, partial casts with thicker outer walls at 60° demonstrated even higher accuracy than the full-arch casts [32]. While these results are not directly comparable to the present in vitro study, since the former compared full- and partial-arch casts, they suggest that printing partial arches may be preferable when possible. It remains challenging to compare results across 3D printing studies, as accuracy is determined by a combination of factors, including the specific printer, the type of additive manufacturing technology, and the resin used.

The second variable analyzed in this study was the use of a hollow base design for the printed casts, with internal shell thicknesses of either 2 mm or 4 mm. To date, no studies have evaluated the accuracy of hollow, full-arch treatment casts printed with varying internal wall thicknesses, making direct comparisons and conclusions with existing literature challenging. Nevertheless, Shin et al. assessed different internal wall thicknesses in anterior and posterior partial casts fabricated using an SLA printer. Their results included the following trueness values: anterior, 0° orientation, 1 mm thickness (52.8 ± 8.7 μm); anterior, 60° orientation, 1 mm (50.9 ± 7.1 μm); posterior, 0° orientation, 1 mm (62.7 ± 9.7 μm); and posterior, 60° orientation, 1 mm (52.8 ± 8.7 μm). These data led the authors to suggest that increased outer wall thickness is associated with greater trueness [32]. Conversely, Revilla-León et al. compared various hollow base designs—hollow cast (H group), honeycomb-structure cast (HC group), and solid cast (S group)—using a material jetting printer (J720 Dental; Stratasys). They observed statistically significant differences in 3D discrepancy, with the H group (34.00 ± 45.00 μm) outperforming both the HC group (58.00 ± 67.25 μm) and the S group (53.00 ± 73.25 μm) [49]. In the present study, the selection of 2 mm and 4 mm external shell thicknesses was intentional and based on both practical laboratory protocols and recommendations for 3D-printed dental models. A 2 mm shell thickness is generally considered the minimum required for adequate mechanical strength and dimensional stability of hollow printed casts according to digital dentistry guidelines and manufacturer instructions, whereas a 4 mm shell is commonly chosen when additional rigidity is needed, particularly for full-arch models or casts that will undergo repeated handling.

In addition to the type of AM technology, the variables evaluated in this study—print orientation and shell thickness—represent key parameters that laboratory technicians and dental clinicians must consider when fabricating 3D-printed casts. These parameters are highly relevant from an economic standpoint, as they influence both manufacturing time and material consumption. Desktop SLA and DLP printers are generally more affordable and compact compared with industrial Multijet printers. However, multiple studies have reported that industrial printers offer superior resolution and printing speed [50]. Consequently, it is anticipated that future advancements in desktop printer technology will continue to enhance the fit and efficiency of 3D-printed dental casts.

The present study has several limitations. Only a single AM technology and one dental printer were evaluated, with the use of a single type of resin and a standardized post-processing protocol. The range of print orientations assessed was limited; a fully solid cast design was not included, and only one layer thickness was tested. Although the printer employed in this study is widely used in dental practice, more advanced models are now available, and the results obtained may vary with different equipment. Further in vitro studies are required to systematically compare these parameters across various types of 3D printers. Manufacturing accuracy in this study was assessed using two measurement approaches: discrepancies across the overall cast and discrepancies at specific points. However, the optimal measurement method remains unclear, and additional studies are needed to compare and validate these approaches. Another limitation is that the gold standard for evaluating the trueness of AM casts—coordinate measuring machine (CMM)-type probing systems—was not used, as the probe does not adapt adequately to the complex geometry of dental casts [39,42,51,52,53,54,55,56,57,58]. Therefore, extraoral scanning of the printed casts was employed. Additional research is necessary to investigate all factors influencing the accuracy of diagnostic and treatment casts and to establish clear manufacturing protocols based on the trinomial of AM technology, 3D printer, and resin type.

Although standardized printing protocols for each combination of AM technology, printer type, and resin have not yet been fully established for all 3D printing applications in dentistry, the practical implications of this study are clear. Under controlled conditions—using the same additive manufacturing technology, printer, resin, and post-processing protocol—treatment casts with high accuracy can be produced by employing a 2 mm shell thickness and a 20° print orientation.

## 5. Conclusions

Within the limitations of this study, the following conclusions can be drawn:

1. For casts printed using an SLA 3D printer intended for fixed dental prostheses, print orientation did not appear to influence the overall accuracy (trueness and precision) of the casts under the conditions described.
2. The shell thickness of the master casts, as tested with the SLA printer, did not affect the overall accuracy. However, when analyzing specific reference points, casts with a 2 mm shell thickness demonstrated better trueness than those with a 4 mm thickness.
3. All printed casts achieved trueness values ranging from 73.9 µm to 193.4 µm, which is considered clinically acceptable.

## Figures and Tables

**Table 1:** The 36 points marked at critical locations on each cast.

Tooth	Points
17	14 (palatal cusp), 15 (buccal cusp), 19 (palatal cusp), 20 (buccal cusp)
16	13 (palatal mesial cusp), 16 (buccal mesial cusp), 21 (buccal distal cusp), 22 (palatal mesial cusp), 23 (buccal finish line), 24 (palatal finish line)
15	12 (palatal cusp), 17 (buccal cusp)
14	11 (palatal cusp), 18 (buccal cusp)
13	10
12	9
11	8
21	6 (incisal edge), 26 (buccal finish line), 27 (palatal finish line)
22	7
23	5 (incisal edge), 25 (buccal finish line), 28 (palatal finish line)
24	4 (palatal cusp), 36 (buccal cusp)
25	3 (palatal cusp), 35 (buccal cusp)
26	2 (palatal cusp), 31 (buccal cusp), 32 (buccal cusp), 33 (palatal cusp)
27	1 (palatal cusp), 29 (buccal cusp), 30 (buccal cusp), 34 (palatal cusp)

**Table 2:** Descriptive statistics for minimum deviation (minDV), maximum deviation (maxDV), and root mean square (RMS) error calculations among the tested groups.

Groups	minDV	maxDV	RMS
Group 1	(−0.342 ± 0.0741)	(0.23 ± 0.0904)	(0.133 ± 0.0383)
Group 2	(−0.29 ± 0.0996)	(0.168 ± 0.079)	(0.116 ± 0.0421)
Group 3	(−0.324 ± 0.074)	(0.189 ± 0.0468)	(0.126 ± 0.0273)
Group 4	(−0.299 ± 0.0566)	(0.195 ± 0.0681)	(0.127 ± 0.0236)
Group 5	(−0.31 ± 0.0545)	(0.187 ± 0.0592)	(0.133 ± 0.0191)
Group 6	(−0.375 ± 0.0994)	(0.273 ± 0.137)	(0.151 ± 0.0424)

minDV: minimum deviation value (mm) [overall mean ± SD]; maxDV: maximum deviation value (mm) [overall mean ± SD]; RMS: root mean square error (mm) [overall mean ± SD].

**Table 3:** Kruskal–Wallis test results for differences in trueness at reference points according to external shell thickness.

Groups	RMS (*p*-Value)
G1 > G4	TDabs2 (*p* = 0.0209), TDabs3 (*p* = 0.0120), TDabs5 (*p* = 0.0005), TDabs6 (*p* = 0.0061), TDabs7 (*p* = 0.0047), TDabs8 (*p* = 0.0463), TDabs12 (*p* = 0.0061), TDabs35 (*p* = 0.0327)
G1 < G4	TDabs1 (*p =* 0.0130)
G2 > G5	TDabs2 (*p* = 0.0008), TDabs3 (*p* = 0.0004), TDabs5 (*p* < 0.0001), TDabs6 (*p* = 0.0012), TDabs8 (*p* = 0.0165), TDabs12 (*p* < 0.0001), TDab35 (*p* = 0.0039), TDab36 (*p* = 0.0350)
G2 < G5	TDabs25 (*p* = 0.0042)
G3 > G6	TDabs2 (*p* = 0.0350), TDabs3 (*p* = 0.0079), TDabs5 (*p* = 0.0032), TDabs6 (*p* = 0.0047), TDabs7 (*p* = 0.0032), TDabs8 (*p* = 0.0209), TDabs11 (*p* = 0.0012), TDabs12 (*p* = 0.0043), TDabs13 (*p* = 0.0032), TDabs24 (*p* = 0.0403), TDab35 (*p* = 0.0282)
G3 < G6	TDabs1 (*p* = 0.0079), TDabs25 (*p* = 0.0350)
G3 > G5 > G6	TDabs2 (*p* = 0.0357)

**Table 4:** Descriptive statistics and Kruskal–Wallis test results for differences in trueness at reference points on abutment teeth according to external shell thickness.

Groups	RMS	X axis	Y Axis	Z Axis
G1 > G4	TDabs5 (*p* = 0.0005), TDabs6 (*p* = 0.0061)	Xabs5 (*p* = 0.0003), Xabs6 (*p* = 0.0078),	Yabs5 (*p* = 0.0003), Yabs6 (*p* = 0.0066) Yabs7 (*p* = 0.0046) Yabs13 (*p* = 0.0395) Yabs28 (*p* = 0.037)	Zabs5 (*p =* 0.0003), Zabs6 (*p =* 0.0066) Zabs7 (*p* < 0.001) Zabs13 (*p* = 0.0376)
G1 < G4	TDabs7 (*p* = 0.006)	Xabs7 (*p* = 0.0038) Xabs13 (*p* = 0.0293) Xabs28 (*p* = 0.049)		Zabs28 (*p* = 0.047)
G2 > G5	TDabs5 (*p* < 0.001), TDabs6 (*p* < 0.001)	Xabs5 (*p* < 0.001), Xabs6 (*p* < 0.001)	Yabs5 (*p* < 0.001), Yabs6 (*p* < 0.001)	Zabs5 (*p* < 0.001), Zabs6 (*p* < 0.001)
G2 < G5	TDabs25 (*p* < 0.001)	Xabs25 (*p* < 0.001)	Yabs25 (*p* < 0.001)	Zabs25 (*p* < 0.001)
G3 > G6	TDabs5 (*p* < 0.001), TDabs6 (*p* < 0.001) TDabs7 (*p* < 0.001) TDabs13 (*p* < 0.001) TDabs24 (*p* = 0.0403)	Xabs5 (*p* < 0.001), Xabs6 (*p* < 0.001) Xabs7 (*p* < 0.001) Xabs13 (*p* < 0.01) Xabs24 (*p* = 0.0403)	Yabs5(*p* < 0.001), Yabs6 (*p* < 0.001) Yabs7 (*p* < 0.001) Yabs13 (*p* < 0.001) Yabs24 (*p* = 0.0403) Yabs25 (*p* = 0.035)	Zabs5 (*p* < 0.001), Zabs6 (*p* < 0.001) Zabs13 (*p* < 0.001) Zabs24 (*p* = 0.0403)
G3 < G6	TDabs25 (*p* = 0.035)	Xabs25 (*p* = 0.035)		Zabs25 (*p* = 0.035)

## Data Availability

The original contributions presented in this study are included in the article. Further inquiries can be directed to the corresponding author.
